# Supplementary material for: Modeling glioblastoma heterogeneity as a dynamic network of cell states
Source: Mol Syst Biol. 2021 Sep 16;17(9):e10105. doi: 10.15252/msb.202010105 (PMC8444284; doi:10.15252/msb.202010105)
Supplement: Supplementary file 6 — Source Data for Figure 5 [file MSB-17-e10105-s004.zip › Figure5A_sourcedata/GSEA_3017/nosigdb_stateD.GseaPreranked.1622795010397/FELDMAN_2020_NEURAL G0.html]

Details for gene set FELDMAN\_2020\_NEURAL G0[GSEA]

|  || Dataset | state23017 |
| Phenotype | NoPhenotypeAvailable |
| Upregulated in class | na\_neg |
| GeneSet | FELDMAN\_2020\_NEURAL G0 |
| Enrichment Score (ES) | -0.50362855 |
| Normalized Enrichment Score (NES) | -2.8492048 |
| Nominal p-value | 0.0 |
| FDR q-value | 0.0 |
| FWER p-Value | 0.0 |
Table: GSEA Results Summary

  

Fig 1: Enrichment plot: FELDMAN\_2020\_NEURAL G0      
 Profile of the Running ES Score & Positions of GeneSet Members on the Rank Ordered List

  

| PROBE | GENE SYMBOL | GENE\_TITLE | RANK IN GENE LIST | RANK METRIC SCORE | RUNNING ES | CORE ENRICHMENT || 1 | PTTG1 |  |  | 14 | 0.490 | -0.0049 | No |
| 2 | PPP1R14B |  |  | 15 | 0.479 | 0.0019 | No |
| 3 | SQLE |  |  | 25 | 0.421 | 0.0003 | No |
| 4 | HSP90AB1 |  |  | 32 | 0.393 | 0.0008 | No |
| 5 | IDI1 |  |  | 34 | 0.382 | 0.0053 | No |
| 6 | RAB13 |  |  | 43 | 0.367 | 0.0038 | No |
| 7 | BTG1 |  |  | 50 | 0.350 | 0.0037 | No |
| 8 | PPA1 |  |  | 64 | 0.328 | -0.0026 | No |
| 9 | HMGB3 |  |  | 80 | 0.311 | -0.0109 | No |
| 10 | SOX4 |  |  | 91 | 0.298 | -0.0151 | No |
| 11 | LDHA |  |  | 92 | 0.297 | -0.0109 | No |
| 12 | MARCKS |  |  | 96 | 0.295 | -0.0093 | No |
| 13 | OLIG2 |  |  | 98 | 0.293 | -0.0060 | No |
| 14 | TROAP |  |  | 109 | 0.283 | -0.0104 | No |
| 15 | TMEM158 |  |  | 120 | 0.275 | -0.0149 | No |
| 16 | BIRC5 |  |  | 130 | 0.271 | -0.0187 | No |
| 17 | S100A16 |  |  | 142 | 0.262 | -0.0242 | No |
| 18 | TUBA1A |  |  | 148 | 0.259 | -0.0248 | No |
| 19 | INSIG1 |  |  | 150 | 0.258 | -0.0220 | No |
| 20 | MKI67 |  |  | 206 | -0.254 | -0.0647 | No |
| 21 | PLD3 |  |  | 240 | -0.258 | -0.0889 | No |
| 22 | TAGLN3 |  |  | 257 | -0.260 | -0.0987 | No |
| 23 | FTL |  |  | 271 | -0.261 | -0.1060 | No |
| 24 | ASAH1 |  |  | 276 | -0.261 | -0.1056 | No |
| 25 | GPM6B |  |  | 314 | -0.265 | -0.1331 | No |
| 26 | TIMP4 |  |  | 315 | -0.265 | -0.1293 | No |
| 27 | FLNA |  |  | 338 | -0.267 | -0.1441 | No |
| 28 | EXOSC8 |  |  | 347 | -0.268 | -0.1470 | No |
| 29 | CARHSP1 |  |  | 405 | -0.274 | -0.1912 | No |
| 30 | TUBB |  |  | 410 | -0.274 | -0.1907 | No |
| 31 | VOPP1 |  |  | 441 | -0.279 | -0.2120 | No |
| 32 | PRDX6 |  |  | 475 | -0.282 | -0.2358 | No |
| 33 | PRSS23 |  |  | 498 | -0.284 | -0.2503 | No |
| 34 | CYR61 |  |  | 555 | -0.292 | -0.2934 | No |
| 35 | RGS6 |  |  | 560 | -0.292 | -0.2926 | No |
| 36 | SIVA1 |  |  | 567 | -0.293 | -0.2935 | No |
| 37 | PAPSS1 |  |  | 621 | -0.304 | -0.3339 | No |
| 38 | PRC1 |  |  | 645 | -0.310 | -0.3489 | No |
| 39 | DNMT1 |  |  | 659 | -0.312 | -0.3554 | No |
| 40 | TMPO |  |  | 688 | -0.317 | -0.3746 | No |
| 41 | KNSTRN |  |  | 690 | -0.317 | -0.3709 | No |
| 42 | S100B |  |  | 705 | -0.320 | -0.3782 | No |
| 43 | KIF4A |  |  | 706 | -0.320 | -0.3737 | No |
| 44 | ACTN1 |  |  | 729 | -0.324 | -0.3876 | No |
| 45 | IGFBP7 |  |  | 733 | -0.326 | -0.3855 | No |
| 46 | PON2 |  |  | 738 | -0.328 | -0.3842 | No |
| 47 | TMSB4X |  |  | 742 | -0.329 | -0.3821 | No |
| 48 | CDC20 |  |  | 793 | -0.341 | -0.4194 | No |
| 49 | NDFIP1 |  |  | 805 | -0.346 | -0.4238 | No |
| 50 | H2AFX |  |  | 810 | -0.347 | -0.4223 | No |
| 51 | SLC44A1 |  |  | 814 | -0.348 | -0.4199 | No |
| 52 | CENPM |  |  | 818 | -0.349 | -0.4175 | No |
| 53 | MAD2L1 |  |  | 819 | -0.350 | -0.4125 | No |
| 54 | MYH9 |  |  | 820 | -0.350 | -0.4076 | No |
| 55 | PRKDC |  |  | 871 | -0.363 | -0.4446 | No |
| 56 | HEY1 |  |  | 882 | -0.366 | -0.4478 | No |
| 57 | CKAP2 |  |  | 900 | -0.375 | -0.4568 | No |
| 58 | TK1 |  |  | 902 | -0.375 | -0.4524 | No |
| 59 | COTL1 |  |  | 907 | -0.376 | -0.4504 | No |
| 60 | TUBB4B |  |  | 916 | -0.380 | -0.4518 | No |
| 61 | USP1 |  |  | 930 | -0.386 | -0.4573 | No |
| 62 | TMEM237 |  |  | 953 | -0.396 | -0.4702 | No |
| 63 | DCBLD2 |  |  | 957 | -0.397 | -0.4671 | No |
| 64 | WSB1 |  |  | 961 | -0.398 | -0.4640 | No |
| 65 | MORC4 |  |  | 987 | -0.408 | -0.4793 | No |
| 66 | AKAP12 |  |  | 994 | -0.412 | -0.4785 | No |
| 67 | MOXD1 |  |  | 996 | -0.412 | -0.4736 | No |
| 68 | CDH2 |  |  | 1007 | -0.418 | -0.4761 | No |
| 69 | CCDC34 |  |  | 1013 | -0.421 | -0.4743 | No |
| 70 | CMC2 |  |  | 1017 | -0.425 | -0.4709 | No |
| 71 | MIS18A |  |  | 1020 | -0.427 | -0.4665 | No |
| 72 | TPX2 |  |  | 1034 | -0.437 | -0.4713 | No |
| 73 | EDNRB |  |  | 1042 | -0.440 | -0.4710 | No |
| 74 | SMC2 |  |  | 1062 | -0.452 | -0.4806 | No |
| 75 | DEPDC1 |  |  | 1074 | -0.457 | -0.4834 | No |
| 76 | ZWINT |  |  | 1099 | -0.467 | -0.4970 | Yes |
| 77 | DLG1 |  |  | 1100 | -0.469 | -0.4904 | Yes |
| 78 | SAT1 |  |  | 1109 | -0.473 | -0.4905 | Yes |
| 79 | ARHGAP29 |  |  | 1121 | -0.487 | -0.4928 | Yes |
| 80 | SPARC |  |  | 1127 | -0.493 | -0.4901 | Yes |
| 81 | PCNA |  |  | 1142 | -0.504 | -0.4948 | Yes |
| 82 | VRK1 |  |  | 1143 | -0.505 | -0.4876 | Yes |
| 83 | TTYH1 |  |  | 1144 | -0.505 | -0.4805 | Yes |
| 84 | FTH1 |  |  | 1152 | -0.514 | -0.4791 | Yes |
| 85 | NUSAP1 |  |  | 1154 | -0.515 | -0.4727 | Yes |
| 86 | GMNN |  |  | 1155 | -0.516 | -0.4654 | Yes |
| 87 | RRM1 |  |  | 1166 | -0.534 | -0.4663 | Yes |
| 88 | ORC6 |  |  | 1179 | -0.551 | -0.4686 | Yes |
| 89 | CENPU |  |  | 1182 | -0.555 | -0.4624 | Yes |
| 90 | CDCA3 |  |  | 1187 | -0.563 | -0.4578 | Yes |
| 91 | ASPM |  |  | 1189 | -0.567 | -0.4507 | Yes |
| 92 | FABP7 |  |  | 1190 | -0.568 | -0.4426 | Yes |
| 93 | NUF2 |  |  | 1194 | -0.574 | -0.4371 | Yes |
| 94 | DHFR |  |  | 1197 | -0.576 | -0.4306 | Yes |
| 95 | CKS1B |  |  | 1198 | -0.577 | -0.4224 | Yes |
| 96 | CLU |  |  | 1207 | -0.592 | -0.4208 | Yes |
| 97 | CST3 |  |  | 1209 | -0.595 | -0.4132 | Yes |
| 98 | GTSE1 |  |  | 1210 | -0.602 | -0.4047 | Yes |
| 99 | CLSPN |  |  | 1212 | -0.605 | -0.3970 | Yes |
| 100 | PLOD2 |  |  | 1215 | -0.615 | -0.3900 | Yes |
| 101 | BEX1 |  |  | 1216 | -0.615 | -0.3813 | Yes |
| 102 | DUT |  |  | 1223 | -0.633 | -0.3774 | Yes |
| 103 | SMC4 |  |  | 1224 | -0.638 | -0.3684 | Yes |
| 104 | CKS2 |  |  | 1226 | -0.641 | -0.3602 | Yes |
| 105 | ANLN |  |  | 1229 | -0.646 | -0.3527 | Yes |
| 106 | HOPX |  |  | 1240 | -0.672 | -0.3517 | Yes |
| 107 | CENPA |  |  | 1243 | -0.679 | -0.3438 | Yes |
| 108 | TAGLN2 |  |  | 1247 | -0.698 | -0.3364 | Yes |
| 109 | LYPD1 |  |  | 1248 | -0.698 | -0.3266 | Yes |
| 110 | AURKB |  |  | 1250 | -0.700 | -0.3175 | Yes |
| 111 | CCNA2 |  |  | 1255 | -0.716 | -0.3108 | Yes |
| 112 | UBE2T |  |  | 1256 | -0.719 | -0.3006 | Yes |
| 113 | HMGB2 |  |  | 1263 | -0.736 | -0.2952 | Yes |
| 114 | LIMCH1 |  |  | 1266 | -0.747 | -0.2864 | Yes |
| 115 | AURKA |  |  | 1271 | -0.763 | -0.2790 | Yes |
| 116 | TYMS |  |  | 1272 | -0.770 | -0.2681 | Yes |
| 117 | ID3 |  |  | 1276 | -0.785 | -0.2595 | Yes |
| 118 | SPP1 |  |  | 1277 | -0.787 | -0.2484 | Yes |
| 119 | IFITM3 |  |  | 1279 | -0.812 | -0.2377 | Yes |
| 120 | ESCO2 |  |  | 1283 | -0.846 | -0.2283 | Yes |
| 121 | CALM2 |  |  | 1286 | -0.878 | -0.2176 | Yes |
| 122 | LGALS3 |  |  | 1287 | -0.878 | -0.2052 | Yes |
| 123 | MALAT1 |  |  | 1289 | -0.884 | -0.1935 | Yes |
| 124 | LRRN3 |  |  | 1293 | -0.932 | -0.1829 | Yes |
| 125 | SPC25 |  |  | 1294 | -0.932 | -0.1697 | Yes |
| 126 | RRM2 |  |  | 1295 | -0.933 | -0.1565 | Yes |
| 127 | KPNA2 |  |  | 1297 | -0.972 | -0.1436 | Yes |
| 128 | CSRP2 |  |  | 1298 | -0.986 | -0.1297 | Yes |
| 129 | IGFBP5 |  |  | 1307 | -1.052 | -0.1215 | Yes |
| 130 | CDK1 |  |  | 1311 | -1.433 | -0.1038 | Yes |
| 131 | UBE2C |  |  | 1312 | -1.466 | -0.0831 | Yes |
| 132 | TOP2A |  |  | 1313 | -1.528 | -0.0615 | Yes |
| 133 | CCL2 |  |  | 1317 | -1.880 | -0.0374 | Yes |
| 134 | HIST1H4C |  |  | 1320 | -2.769 | 0.0000 | Yes |
Table: GSEA details [plain text format]

  

Fig 2: FELDMAN\_2020\_NEURAL G0: Random ES distribution      
 Gene set null distribution of ES for **FELDMAN\_2020\_NEURAL G0**
